# Preoperative assessment of tumor consistency and gross total resection in pituitary adenoma: Radiomic analysis of T2-weighted MRI and interpretation of contributing radiomic features

**DOI:** 10.1016/j.bas.2025.104237

**Published:** 2025-03-13

**Authors:** Martin Černý, Vojtěch Sedlák, Martin Májovský, Petr Vacek, Kateřina Sajfrídová, Kíra R. Patai, Alexia-Ştefana Mârza, David Netuka

**Affiliations:** a Department of Neurosurgery and Neurooncology, Military University Hospital Prague, Czech Republic; b Faculty of Medicine, Charles University, Prague, Czech Republic; c Department of Radiodiagnostics, Military University Hospital Prague, Czech Republic; d Medical School, University of Pécs, Pécs, Hungary; e Faculty of Medicine, Lucian Blaga University, Sibiu, Romania

**Keywords:** Pituitary adenoma, Radiomics, Magnetic resonance imaging, Tumor consistency, Gross total resection

## Abstract

**Background:**

Preoperative knowledge of tumor consistency and the likelihood of gross total resection (GTR) would greatly benefit planning of pituitary adenoma surgery, however, no reliable methods currently exist.

**Objectives:**

To evaluate the utility of radiomic analysis of MRI for predicting tumor consistency and GTR. To explore the interpretability of contributing radiomic features.

**Methods:**

Patients undergoing first endoscopic surgery for pituitary macroadenomas were included. Tumor consistency was assessed intraoperatively, GTR was assessed based on postoperative MRI. Radiomic features were extracted from axial T2-weighted MRI. Low-variability and highly intercorrelated features were removed. Random Forest Classifiers were optimized using 70 % of patient data and evaluated on the remaining 30 %. Relative feature importance was assessed using the Gini–Simpson index.

**Results:**

542 patients were included. GTR was achieved in 325 (60.0 %) cases, firm tumors were encountered in 122 (22.5 %) cases. There was a significant correlation between GTR and tumor consistency (67.1 % vs. 35.2 %, p < 0.001). 1688 radiomic variables were extracted, 442 were removed due to low variance and 699 due to high intercorrelation. The consistency prediction model achieved an accuracy of 81.6 % and utilized 32 features, GTR prediction model achieved 79.1 % accuracy using 73 features.

**Conclusions:**

Radiomic analysis demonstrated significant potential for preoperative evaluation of pituitary adenomas. Texture and intensity-based features were the primary contributors to consistency prediction. However, the explanation of these features was insufficient. GTR prediction was predominantly driven by shape-related features. Our findings highlight the challenges of linking radiomic features to underlying tissue properties and emphasize the need for cautious interpretation.

## Highlights

• Radiomic analysis demonstrates good accuracy in preoperative assessment of tumor consistency and gross total resection.
• Texture and intensity-based features were the main contributors to consistency prediction, their explainability remains poor.
• GTR prediction was predominantly driven by shape-related features, with tumor width and shortest axis length being the most prominent predictors.
• Understanding the underlying the underlying meaning of radiomic features is essential for the safe use of radiomics in clinical decision making.

## 1 Introduction

Pituitary adenomas (PAs) are common benign tumors originating in the pituitary gland, accounting for approximately 10–15 % of all intracranial neoplasms (Daly and Beckers, 2020). While benign, these tumors can have significant clinical implications, particularly when causing mass effects or abnormal hormone secretion, leading to symptoms that impact a patient's quality of life (Iglesias et al., 2018; Russ et al., 2023). The primary treatment for PAs involves surgical resection, typically performed using a minimally invasive endoscopic endonasal approach (Banskota and Adamson, 2021; Jane et al., 2000). Certain tumor characteristics, including tumor volume, suprasellar extension, cavernous sinus invasion, and tumor consistency, can pose challenges during surgery (Heffernan et al., 2022; Chowdhury et al., 2014; Naganuma et al., 2002).

Tumor consistency refers to the physical properties of the tumor tissue, specifically its firmness or texture, which can vary significantly among PAs. Tumors with a soft consistency are generally easier to resect, often requiring only basic surgical tools, while fibrous or hard tumors present a greater challenge, necessitating more complex surgical maneuvers such as extracapsular dissection or an extended endonasal approach, and dedicated tools such as ultrasonic aspirator (Cappelletti et al., 2019). Tumor consistency is a critical factor influencing the outcome of the surgery, with harder tumors associated with a higher likelihood of incomplete resection and postoperative complications, including transient diabetes insipidus, cerebrospinal fluid leak, and cranial nerve paresis (Naganuma et al., 2002; Guerra et al., 2025). Therefore, accurately predicting tumor consistency before surgery could greatly enhance surgical planning by informing decisions on the appropriate surgical approach and tools, potentially reducing the risk of residual tumor and complications (Snow et al., 1986).

Recent efforts have aimed to develop reliable methods for preoperative assessment of pituitary adenoma consistency using various MRI modalities, however, with mixed results (Černý et al., 2022). Table 1 presents an overview of published studies and their findings.

**Table 1:** Overview of published studies investigating the correlation between MRI characteristics and pituitary adenoma consistency. The table summarizes findings from various imaging modalities, including T2-weighted MRI, diffusion-weighted imaging (DWI), dynamic contrast-enhanced studies, and magnetic resonance elastography (MRE). Reported correlations between imaging features and tumor consistency vary, with some studies indicating hypointensity, hyperintensity, increased or decreased diffusivity in firm tumors, while others report no significant association.

Imaging modality	Conclusion	Year	Author	N	% of firm tumors
T2-weighted MRI	Firm tumors hypointense	2015	Wei et al. (Wei et al., 2015)	38	21
		2020	Chen et al. (Chen et al., 2020)	191	21
		2020	Yun et al. (Yun et al., 2020)	50	18
	Firm tumors hyperintense	2006	Pierallini et al. (Pierallini et al., 2006)	22	18
	No correlation	2019	Mastorakos et al. (Mastorakos et al., 2019)	196	24
		2020	Guinto-Nishimura et al. (Guinto-Nishimura et al., 2020)	26	23
		2021	Li et al. (Li et al., 2021)	55	35
DWI	Higher diffusivity in firm tumors	2006	Pierallini et al. (Pierallini et al., 2006)	22	18
		2013	Mohamed et al. (Mohamed and Abouhashem, 2013)	30	10
		2014	Thomas et al. (Thomas et al., 2014)	31	19
		2017	Alashwah et al. (Alashwah et al., 2017)	20	20
	Lower diffusivity in firm tumors	2015	Wei et al. (Wei et al., 2015)	38	21
		2019	Alimohamadi et al. (Alimohamadi et al., 2014)	16	13
		2020	Rutland et al. (Rutland et al., 2020)	13	15
		2021	Ding et al. (Ding et al., 2021)	183	19
	No correlation	2020	Guinto-Nishimura et al. (Guinto-Nishimura et al., 2020)	26	23
		2021	Kamimura et al. (Kamimura et al., 2021)	49	31
Dynamic contrast enhancement studies	Firm tumor display higher degree of enhancement	1998	Iuchi et al. (Iuchi et al., 1998)	26	19
	Firm tumors display less enhancement	2017	Romano et al. (Romano et al., 2017)	21	29
	Mosaic enhancement pattern associated with soft tumors	2014	Yamamoto et al. (Yamamoto et al., 2014)	29	17
MRE/vMRE	Shear stiffness higher in firm tumors	2016	Sakai et al. (Sakai et al., 2016)	11	9
		2016	Hughes et al. (Hughes et al., 2016)	10	40
		2021	Cohen-Cohen et al. (Cohen-Cohen et al., 2021)	38	5
	No correlation	2021	Lagerstrand et al. (Lagerstrand et al., 2021)	10	10

Radiomics approaches involve extracting a variety of quantitative features from medical imaging to discern patterns correlated with tumor characteristics. These methods can include metrics such as signal intensity, texture features, and shape characteristics derived from MRI scans (Cuocolo et al., 2020; Fan et al., 2019; Rui et al., 2019; Wan et al., 2022; Wang et al., 2021; Zeynalova et al., 2019).

One of the major criticisms of radiomics is its inherent lack of explainability (Horvat et al., 2024). Most radiomic models function as “black box” systems, where the relationship between the imaging features and the final prediction is often unclear (Muhammad and Bendechache, 2024). To improve the utility of radiomics, there is a pressing need for explainability of model decisions (Ibrahim et al., 2021; Varriano et al., 2022). It is crucial to understand what individual radiomic features represent, how they correlate with observable radiographic characteristics, and how they connect to underlying pathological processes (Zhang et al., 2023). The aim of this study is to evaluate the utility of radiomic analysis of preoperative T2-weighted MRI for predicting tumor consistency and GTR likelihood in pituitary adenoma and to explore the interpretability of contributing radiomic features.

## 2 Methods

### 2.1 Patient data

**Fig. 1:**
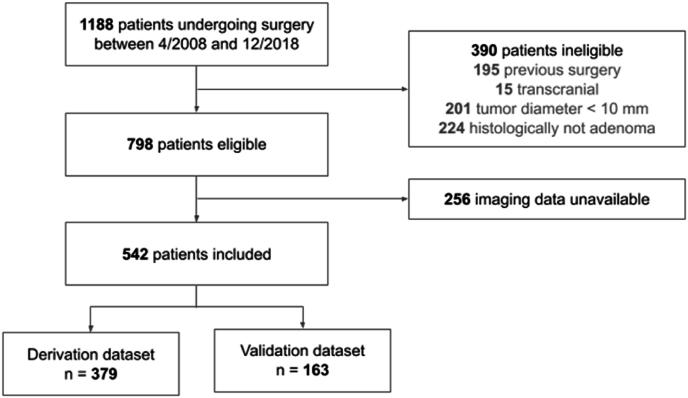
Flowchart of patient inclusion and dataset assignment. Patients undergoing first endoscopic surgery for histologically confirmed pituitary adenoma with tumor diameter ≥10 mm were included from a prospectively managed database. Patients without the required imaging data (contrast-enhanced coronal T1-weighted and axial T2-weighted MRI scans) not older than one month before the surgery were excluded. The remaining eligible patients were randomly split into derivation (70 %) and validation (30 %) datasets for radiomic analysis.

We reviewed patients who underwent first endoscopic surgery for a histologically confirmed pituitary macroadenoma (tumor diameter ≥10 mm) from a prospectively managed database of surgeries performed in our institution for sellar lesions between April 2008 and December 2018. GTR was assessed based on the presence of a tumor residuum on the early postoperative MRI scan performed routinely before discharge (POD 3–5). The tumor consistency was assessed by the surgeon as either soft or firm and recorded directly after the surgery. A soft tumor was defined as one that could be aspirated or easily removed using standard suction and curettes without requiring additional mechanical fragmentation. A firm tumor required the use of additional surgical techniques, including extracapsular dissection, piecemeal removal, or ultrasonic aspiration due to resistance to standard suction. For tumors with cystic components, only the solid portion was considered in consistency assessment. The presence of tumor hemorrhage (apoplexy) was extracted retrospectively from the operative notes for all cases. Fig. 1 presents a flowchart of patient inclusion and assignment.

### 2.2 Radiomic features extraction

**Fig. 2:**
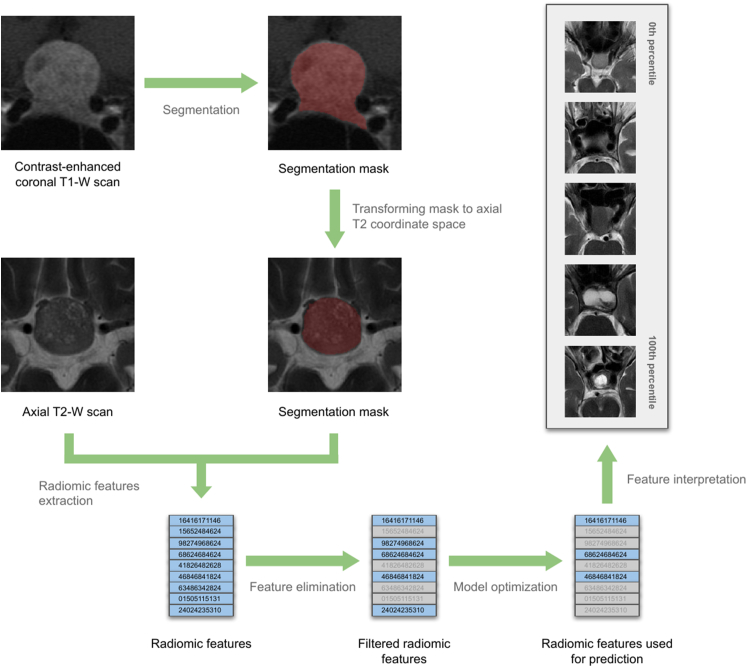
A schematic overview of the radiomic pipeline for predicting tumor consistency and gross total resection. First, automated segmentation of the tumor from the contrast-enhanced coronal T1-weighted scan is performed, followed by the transformation of segmentation masks into the axial T2-weighted scan coordinate space. Radiomic features are then extracted from the segmented tumor region. Feature elimination is performed to reduce redundancy and retain the most informative features. A machine learning model is optimized based on the selected features, radiomic features contributing to the prediction are identified and their relative importance is assessed quantitatively. Finally, feature interpretation is conducted to understand the relationship between predictive features and tumor characteristics, here demonstrated by five scans representing values for one of the variables (wavelet-LLH_glcm_Correlation) ordered from the lowest to the highest encountered value.

For each patient, a contrast-enhanced coronal T1-weighted and an axial spin echo T2-weighted MRI scans were identified manually. A patient was excluded if no scans not older than one month before the surgery were available. Scans acquired with various in-house and extramural imaging protocols on various machines were used. The coronal scans were automatically segmented using the pituitary adenoma 3D segmentation model developed in our previous work (Černý et al., 2024). The segmented tumor volume was transformed into the coordinate space of the axial T2-weighted scan. Radiomic features were extracted from the previously segmented tumor area using PyRadiomics library v3.1.0 (van Griethuysen et al., 2017). Fig. 2 presents the data extraction process. Extracted radiomic features are available as Appendix 1.

### 2.3 Feature selection

Feature selection was conducted to remove redundant features. Features with low variability were excluded using a variance threshold of 0.01. Features exhibiting high intercorrelation were identified through a pairwise Pearson correlation matrix, with a threshold of 0.9 applied to exclude highly correlated variables.

### 2.4 Outcomes

The primary outcome of this study were the relative feature importances of radiomic features for the prediction of GTR and tumor consistency. The secondary outcomes included.

● correlation between GTR and tumor consistency,
● correlation between the presence of hemorrhage and tumor consistency, and
● accuracy of the prediction models on the subpopulation of hemorrhagic tumors.

### 2.5 Statistical analysis

The dataset was divided into derivation and validation subsets with 70 % used for model optimization and the remaining 30 % allocated for validation. A Random Forest Classifier was optimized for both classification tasks (firm tumor consistency and GTR). Feature importances were assessed by quantifying the reduction in uncertainty at each decision tree split, using the *Gini–Simpson index*, calculated as

$$G=1-\sum_{i=1}^{C}p_{i}^{2}$$

where *p*_*i*_ represents the proportion of samples belonging to class *i* at a given node, and *C* is the number of classes (Gini, 1912). All feature variables were then ranked according to their importance scores. The model's accuracy was evaluated for each subset of top N features, and the optimal number of features, defined by the highest achieved accuracy, was identified.

The correlation between GTR and tumor consistency was assessed using the Chi-square test, comparing the proportion of GTR achieved in soft vs. firm tumors. The correlation between hemorrhage and tumor consistency was evaluated using the Chi-square test, comparing the proportion of hemorrhagic vs. non-hemorrhagic tumors classified as soft. Statistical code is available as Appendix 2.

## 3 Results

### 3.1 Data

From the 1188 records in the database, 798 met the inclusion criteria. Required imaging data were available for 542 patients. GTR was achieved in 325 (60.0 %) cases, firm tumors were encountered in 122 (22.5 %) cases, tumor hemorrhage was encountered in 30 (5.5 %) cases. There were 266 (49.0 %) of male patients, the mean age at the time of surgery was 53.5 ± 15.2 years. The mean tumor diameter was 22.1 ± 10.9 mm, 206 (38.0 %) of the tumors were hormonally active. Patient baseline characteristics are summarized in Table 2. Characteristics of the imaging data are summarized in Table 3.

**Table 2:** Patient baseline characteristics in the whole dataset and derivation and validation subsets. Differences between the derivation and validation subsets are presented as p-values and were calculated using a two-sample *t*-test for continuous variables, and a chi-square test for categorical variables.

	All data	Derivation dataset	Validation dataset	Difference der/val
**N**	542	379	163	
**Age***years, mean ± SD*	53.54 ± 15.17	53.89 ± 15.54	52.73 ± 14.28	p = 0.415
**Male sex***n (%)*	266 (49.0)	182 (48.02 %)	84 (51.53 %)	p = 0.512
**Tumor diameter***mm, mean ± SD*	22.11 ± 10.89	21.62 ± 10.28	23.23 ± 12.13	p = 0.116
**NFPA***n (%)*	336 (62.0)	232 (61.2)	104 (63.8)	p = 0.636
**Firm***n (%)*	122 (22.5)	85 (22.4)	37 (22.7)	p = 0.966
**GTR***n (%)*	325 (60.0)	227 (59.9)	98 (60.1)	p = 0.963
**Hemorrhage***n (%)*	30 (5.5)	22 (5.8)	8 (4.9)	p = 0.831

**Table 3:** Imaging data characteristics. Differences between the derivation and validation subsets are presented as p-values and were calculated using a two-sample *t*-test; TR - repetition time, TE - echo time, FOV - field of view.

	All data	Derivation dataset	Validation dataset	Difference der/val
**TR***ms, mean (± SD)*	6022.00 ± 915.42	6021.03 ± 858.26	6024.27 ± 1039.10	p = 0.972
**TE***ms, mean (± SD)*	110.65 ± 14.42	110.30 ± 14.49	111.48 ± 14.25	p = 0.379
**FOV***cm, mean (± SD)*	240.57 ± 6.72	240.93 ± 5.46	239.73 ± 8.97	p = 0.130
**Slice spacing***mm, mean (± SD)*	5.82 ± 0.50	5.82 ± 0.49	5.81 ± 0.52	p = 0.851
**Slice thickness***mm, mean (± SD)*	4.89 ± 0.35	4.89 ± 0.34	4.87 ± 0.37	p = 0.656
**Pixel spacing***mm, mean (± SD)*	0.48 ± 0.07	0.48 ± 0.07	0.47 ± 0.08	p = 0.410
**Resolution in pixels***n, mean (± SD)*	508.90 ± 59.05	505.96 ± 43.69	515.73 ± 84.39	p = 0.163
**# of averages***n, mean (± SD)*	1.55 ± 0.23	1.55 ± 0.25	1.56 ± 0.20	p = 0.659
**Pixel bandwidth** *Hz, mean (± SD)*	298.79 ± 181.55	298.57 ± 172.78	299.29 ± 201.36	p = 0.969

### 3.2 Feature extraction and selection

**Fig. 3:**
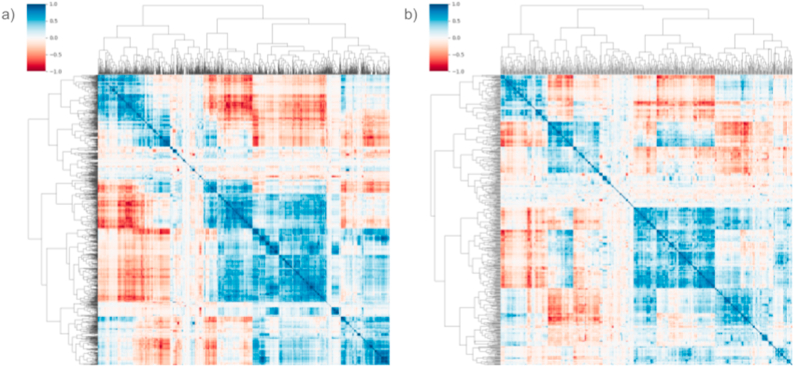
Hierarchically clustered heatmap of the radiomic features correlation matrix a) before and b) after feature elimination. Highly intercorrelated variables (carrying very similar information) appear as intense blue (positive correlation) or intense red (negative correlation). After feature elimination, the portion of intense color points in the heatmap is decreased.

A total of 1688 radiomic variables were extracted for each patient. Subsequently, 442 features were removed due to low variance, and an additional 699 features were excluded based on high intercorrelation, leaving a total of 547 features for further analysis. Fig. 3 shows a hierarchically clustered heatmap of the feature correlation matrix before and after feature elimination.

### 3.3 Classification accuracy

For tumor consistency classification, the highest accuracy (81.6 %) was achieved using 32 radiomic features, with the inclusion of additional features not resulting in improved performance. For the GTR classification, the highest accuracy (79.1 %) was obtained using 73 radiomic features. Table 4 summarizes the top 10 radiomic features for each classifier and their respective importance scores. The full set of features used in both classifiers and their relative importances are available as Appendix 3.

**Table 4:** Summary of top 10 radiomic features for both classifiers and their relative importance scores in predictive models for tumor consistency and gross total resection. The table lists the most significant radiomic features ranked by their relative importance. The composed accuracy for each feature subset is also presented, illustrating their incremental contribution to the model's predictive performance.

	Feature name	Feature importance (%)	Composed accuracy (%)
Firm tumor consistency prediction			
1.	wavelet-LLH_glcm_Correlation	4,92	69,33
2.	wavelet-LLL_glcm_MCC	4,49	74,23
3.	squareroot_firstorder_Maximum	4,05	75,46
4.	original_glcm_DifferenceEntropy	4,03	74,23
5.	wavelet-HHL_firstorder_Skewness	3,82	74,23
6.	wavelet-LHL_glcm_Correlation	3,75	76,07
7.	logarithm_ngtdm_Contrast	3,66	74,23
8.	wavelet-HHH_glcm_ClusterShade	3,51	74,85
9.	wavelet-HHH_firstorder_Skewness	3,33	74,85
10.	wavelet-HLL_firstorder_Maximum	3,23	73,62
**Gross total resection prediction**			
1.	original_shape_Maximum2DDiameterColumn	6,09	66,87
2.	original_shape_LeastAxisLength	3,20	65,03
3.	gradient_gldm_DependenceNonUniformity	3,11	73,01
4.	lbp-2D_gldm_DependenceNonUniformity	2,94	68,10
5.	original_shape_MajorAxisLength	2,87	65,03
6.	exponential_gldm_LargeDependenceEmphasis	2,77	67,48
7.	original_shape_Maximum2DDiameterSlice	2,52	66,26
8.	original_shape_Maximum2DDiameterRow	2,11	70,55
9.	lbp-3D-k_glszm_GrayLevelNonUniformity	2,01	71,78
10.	exponential_gldm_DependenceNonUniformity	1,97	71,78

### 3.4 Secondary outcomes

There was a significant correlation between GTR and tumor consistency (67.1 % in soft tumors vs. 35.2 % in firm tumors, p < 0.001). The correlation between soft tumor consistency and the presence of hemorrhage did not reach statistical significance (26.7 % vs. 22.3 %, p = 0.737). The prediction model's accuracy in the subpopulation of hemorrhagic tumors was 75 % for consistency prediction and 87.5 % for GTR predictions.

## 4 Discussion

The radiomic models achieved a high predictive accuracy of 81.6 % for tumor consistency and 79.1 % for GTR, demonstrating robust generalizability across various in-house and extramural imaging protocols and various machines. Tumor consistency was predominantly predicted by a lower number of texture-based features, such as those related to intensity distribution, homogeneity, and spatial patterns. GTR was influenced by a larger number of features, each with lesser relative importance. Most prominent were size and shape-related features, particularly the tumor's overall dimensions and surface-to-volume ratio. The total prediction accuracy for GTR was lower than for tumor consistency, possibly reflecting the fact that further factors contribute to the surgical outcome that can not be fully captured by preoperative imaging alone (occurrence of intraoperative complications, incorrect intraoperative identification of tumor tissue, surgeon experience).

**Fig. 4:**
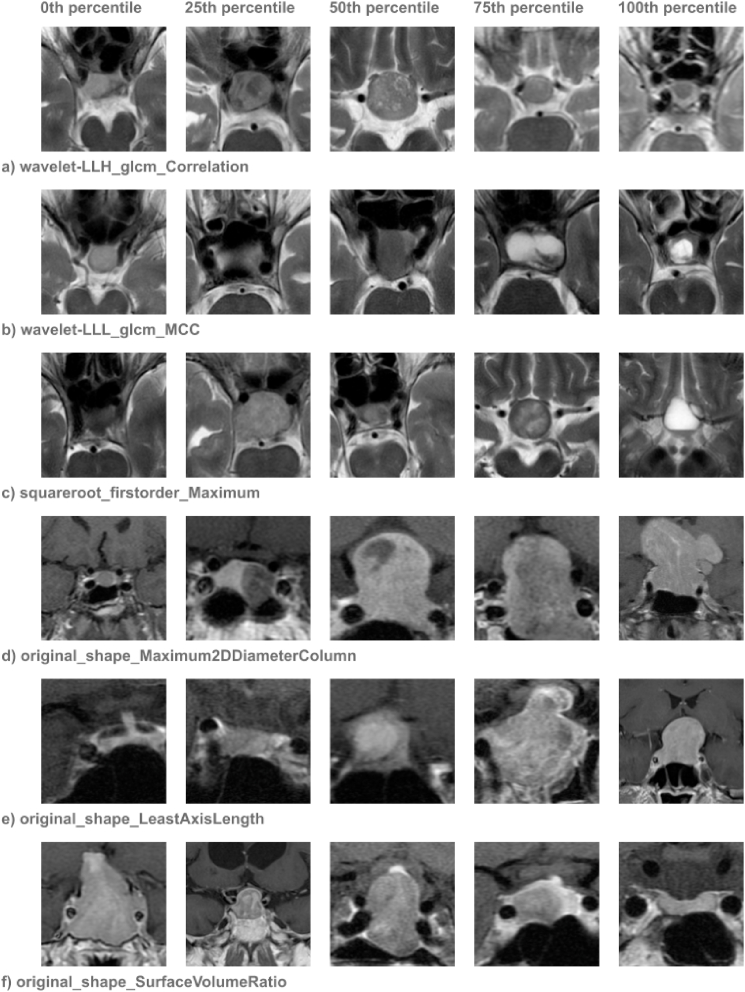
Selected radiomic features and the corresponding scans for the 0th, 25th, 560th, 75th and 100th percentile. Panels a–c represent texture-based and intensity-based features and are demonstrated on axial T2-weighted images, from which they were extracted. Panels d–f represent shape-based features and are demonstrated on coronal contrast-enhanced T1-weighted scans. a) GLCM (gray level correlation matrix) correlation measures homogeneity within the tumor. Tumors with low GLCM correlation (left) exhibit significant intra-tumor heterogeneity, whereas high GLCM correlation represents a solid heterogeneous well-delineated tumor (100th percentile sample). b) Wavelet LLL GLCM MCC quantifies the complexity of textures in the low-pass filtered (smoothed) version of the image. A higher value suggests more intricate and interrelated textures, which typically corresponds to finer, more granular textures. c) First order maximum assesses the maximum intensity value. However, MRI sequences typically lack signal intensity standardization, meaning that absolute intensities can vary. d) Maximum 2D diameter column measures the widest horizontal distance (left to right), providing information about the parasellar growth of the tumor. The widest horizontal distance is a part of the calculation of the Zurich Pituitary Score validated to reliably stratify the likelihood of GTR [62]. e) Least axis length is a shape-based radiomic feature that measures the shortest dimension of the bounding ellipsoid that fits the tumor in three-dimensional space, reflecting its minimal spatial extent. f) Surface volume ratio assesses the relationship between surface area and volume, with lower values corresponding to irregular, jagged surfaces.

In the following text, we discuss the most prominent radiomic features in both models and attempt to understand what these features represent, how they correlate with observable radiographic characteristics, and how they connect to underlying pathological processes. Although it is important to interpret the results and the meaning of the radiomic features, we need to acknowledge that any interpretation is a mere assumption and requires further research and validation. Fig. 4 presents the corresponding scans for the 0th, 25th, 50th, 75th, and 100th percentile for selected radiomic variables.

**Texture-based features** primarily focus on the spatial distribution of intensity and patterns of pixel relationships. They measure the heterogeneity, uniformity, and structural complexity of the tumor. *GLCM (gray level correlation matrix) correlation* measures the correlation between pixel intensities and is related to homogeneity within the tumor. Solid-type tumors, which have a more uniform texture, show higher correlation values (Fig. 4a). *Wavelet LLL (low-pass filtered in all three orthogonal planes) GLCM MCC (Maximal Correlation Coefficient)* quantifies the complexity of textures in the low-pass filtered (smoothed) version of the image using the maximal correlation coefficient derived from the GLCM. A higher value suggests more intricate and interrelated textures, which typically corresponds to finer, more granular textures. (Fig. 4b). *Cluster shade* and *Dependence variance* both assess contrast and texture variability. *Difference entropy* quantifies the randomness of the intensity differences between neighboring pixel pairs. *Large dependence emphasis* quantifies the degree to which larger, homogeneous regions with consistent gray levels dominate in the image. In our dataset, we encountered insignificantly lower *GLCM correlation* (p = 0.051) and significantly higher *LLL GLCM MCC* (p = 0.001) in firm tumors, suggesting that higher heterogeneity is associated with firm tumor consistency. However, these findings are not in accordance with existing literature, where higher heterogeneity is usually explained by more complex internal structures, such as areas of necrosis or calcification and cystic components, typically believed to contribute to softer consistency (Iuchi et al., 1998; Yamamoto et al., 2014). We do not have a sufficient explanation for these conflicting results.

**Intensity-based features** aid in analyzing the brightness and contrast of tumor tissues, providing valuable insights into the tumor's internal composition and density. These features are part of first-order statistics, which describe the basic distribution of pixel intensities without considering their spatial relationships, and provide a way to evaluate how uniformly distributed or concentrated the pixel intensities are across the image. *Square root first order maximum* assesses the maximum intensity value in the tumor (Fig. 4c). *First-order skewness* quantifies the degree of non-gaussian distribution of image intensities (asymmetry in intensity histogram). *Kurtosis* is a statistical measure that describes the "tailedness" or shape of the intensity distribution of pixel intensities and reflects tissue heterogeneity. In our study, *First order maximum* contributed to tumor consistency prediction and firm tumors were associated with higher values, although not significantly (p = 0.056). Literal reports on the relationship between T2 intensity and firm tumor consistency are conflicting, with some finding negative correlation (Chen et al., 2020; Wei et al., 2015; Yun et al., 2020), some finding positive correlation (Pierallini et al., 2006) and some contesting any relationship (Guinto-Nishimura et al., 2020; Li et al., 2021; Mastorakos et al., 2019). Current findings do not support the role of T2 intensity alone as a reliable predictor of tumor consistency (Černý et al., 2022). It is also important to note that most studies do not use absolute signal intensity value but rather relative values normalized to a reference area in white matter or cerebellar peduncles (Chen et al., 2020; Mastorakos et al., 2019) to compensate for the inherent lack of standardization of signal intensity in MRI. This is not possible with the current version of PyRadiomics library, possibly limiting the utility of intensity-based features (van Griethuysen et al., 2017).

**Shape and size-based features** focus on the geometrical dimensions and spatial characteristics of the tumor, providing important information about its overall structure. *Maximum 2D diameter column* (Fig. 4d), *slice, and row* measure the largest tumor dimensions in respective orthogonal planes and all three were among the most important features for GTR prediction. *Maximum 2D diameter column*, in the case of an axial scan the widest horizontal distance (left to right) was the single most important feature. This is well in correspondence with the findings in the literature as tumors with irregular shapes and increased size often present significant challenges during surgery (Erkan et al., 2021; Gondim et al., 2014; Naganuma et al., 2002). Multiple grading systems based on the parasellar and suprasellar extension have been proposed and proven to reliably stratify the likelihood of GTR, endocrinological remision and other clinical outcomes (Hardy and Vezina, 1976; Knosp et al., 1993; Serra et al., 2018; Wilson, 1984). The widest horizontal distance specifically is a part of the calculation of the Zurich Pituitary Score validated to reliably stratify the likelihood of GTR [62]. Unlike the previously discussed two-dimensional radiomic features, *Least axis length* (Fig. 4e) and *Major axis length* measures the shortest and longest dimension of the bounding ellipsoid that fits the tumor in three-dimensional space, more precisely reflecting its extent. *Surface volume ratio* (Fig. 4f) assesses the relationship between surface area and volume, with lower values corresponding to irregular, jagged surfaces.

Overall, the features contributing to GTR could be well explained by intuitive shape-related factors, such as tumor dimensions and surface-to-volume ratio, which are well-documented in the literature. However, the explainability of tumor consistency prediction remains unsatisfactory. Despite achieving a good accuracy in the consistency prediction model on a separate validation subset, the inner workings of this model remain poorly understood, as our attempts to interpret individual features were often conflicting with the existing literature.

The GTR model utilized a notably larger number of features for its predictions (73 vs. 32). One possible explanation is that non-shape related features, such as those influencing consistency, may play a role in GTR prediction. On our dataset, we indeed found a strong association between GTR and tumor consistency (67.1 % in soft tumors vs. 35.2 % in firm tumors, p < 0.001), corresponding to the majority of literature. Alternatively, this can be attributed to volume-confounding effects documented in radiomics (Traverso et al., 2020), possibly leading to shape-related factors influencing texture and intensity-based features.

Looking ahead, radiomics holds great promise for enhancing preoperative tumor assessments, personalizing surgical approaches, complication assessment and patient counseling. However, it is crucial to strive for both the reliability and explainability of radiomic models and caution must be exercised in its clinical application. Only through transparent and interpretable models can we ensure that radiomics truly aids in clinical decision-making and becomes a valuable tool in neurosurgery. Further research and validation are necessary to address the gaps in model understanding and improve their practical utility. It is also necessary to study the underlying histopathological features of the tissue to understand the tissue basis of tumor consistency (Yilmaz et al., 2025).

## 5 Limitations

The study was conducted at a single institution, possibly limiting the generalizability of the findings to other centers with different patient populations or surgical practices.

Variability in MRI scanners, acquisition settings, and contrast administration could have affected the extracted radiomic features and introduced variability into the results.

Signal intensity normalization could not be performed due to the limitations of the software used (van Griethuysen et al., 2017).

The study focused solely on macroadenomas (tumor diameter ≥10 mm).

The study was performed retrospectively, potentially introducing selection bias due to the inclusion of only patients with complete data.

Attempts to explain the role of individual radiomic features, may be incorrect or oversimplified.

## 6 Conclusions

Our study demonstrated the potential of radiomic analysis of preoperative T2-weighted MRI for predicting tumor consistency and the likelihood of GTR in pituitary adenoma surgery. The predictive models achieved accuracies of 81.6 % for tumor consistency and 79.1 % for GTR, highlighting the relevance of texture, size, and shape-related features. While the explainability of the GTR model was satisfactory, aligning well with established anatomical principles, the consistency model presented significant challenges in interpretation. Many of the identified radiomic features lacked clear connections to tumor biology, and some associations contradicted existing literature, underscoring the risk of oversimplification or misinterpretation in feature analysis. These challenges reflect the inherent complexity of linking radiomic features to underlying pathological processes and emphasize the need for caution when attempting to draw medical conclusions from computational models. To ensure that radiomics becomes a reliable tool in clinical practice, future efforts must focus on validating these findings, improving model transparency, and developing robust methods to bridge the gap between computational predictions and clinical decisions. Radiomics shows promise for enhancing surgical planning and personalization in neurosurgery, but achieving transparency and reliability remains a critical challenge for widespread clinical adoption.

## Author contributions

Martin Černý; conceptualized the study, performed the statistical analysis, and drafted the manuscript. Vojtěch Sedlák authored the sections pertaining to magnetic resonance imaging methodology and interpretation. Martin Májovský; and Petr Vacek authored the sections discussing the clinical background and implications of the findings. Kateřina Sajfrídová; was responsible for data management and the extraction of radiomic features. Kíra Patai and Alexia-Ştefana Mârza assisted in the review of clinical cases and the selection and extraction of imaging data. David Netuka supervised and reviewed the work on this article as the senior author. All authors have given their approval of the final version to be published.

## Ethical standards

This study was approved by the institutional ethical committee of Military University Hospital (ref. nr. 108/17-9/2022). Data were anonymized at the time of patient inclusion and treated according to the ethical standards of the Declaration of Helsinki. The requirement of informed consent was waived by the institutional ethical committee because of the retrospective nature of the study and no potential harm to the study participants.

## Use of generative AI

No generative AI tools were used during the preparation of this work.

## Competing interest

All authors declare to have no competing interest.
